# Cancer type, stage and prognosis assessment from pathology reports using LLMs

**DOI:** 10.1038/s41598-025-10709-4

**Published:** 2025-07-26

**Authors:** Rachit Saluja, Jacob Rosenthal, Annika Windon, Yoav Artzi, David J. Pisapia, Benjamin L. Liechty, Mert R. Sabuncu

**Affiliations:** 1 Cornell University, Ithaca, USA; 2 Cornell Tech, New York, USA; 3 Weill Cornell Medicine, New York, USA; 4 Weill Cornell/Rockefeller/Sloan Kettering Tri-Institutional M.D.-Ph.D. Program, New York, USA

**Keywords:** Cancer, Engineering, Health care

## Abstract

Large Language Models (LLMs) have shown significant promise across various natural language processing tasks. However, their application in the field of pathology, particularly for extracting meaningful insights from unstructured medical texts such as pathology reports, remains underexplored and not well quantified. In this project, we leverage state-of-the-art language models, including the GPT family, Mistral models, and the open-source Llama models, to evaluate their performance in comprehensively analyzing pathology reports. Specifically, we assess their performance in cancer type identification, AJCC stage determination, and prognosis assessment, encompassing both information extraction and higher-order reasoning tasks. Based on a detailed analysis of their performance metrics in a zero-shot setting, we developed two instruction-tuned models: **Path-llama3.1-8B** and **Path-GPT-4o-mini-FT**. These models demonstrated superior performance in zero-shot cancer type identification, staging, and prognosis assessment compared to the other models evaluated.

## Introduction

Cancer remains one of the leading causes of death globally^1^. The diagnosis and prognosis of cancer require the collaboration of numerous healthcare professionals, who invest considerable time and expertise to optimize patient outcomes. Despite this effort, current practices lack a cohesive, data-driven approach that could significantly enhance diagnosis and prognosis by leveraging the vast amounts of clinical information available in unstructured text within the medical record, such as pathology reports. Due to time and budget constraints, healthcare professionals are often unable to analyze and extract meaningful insights from the millions of pathology reports generated, which limits their ability to conduct comprehensive analyses for improving patient care.

Pathology reports, in particular, are complex and unstructured medical documents characterized by inconsistent formatting and a high degree of variability^2,3^. These reports often include extensive sections of information that are clinically irrelevant, making them less efficient for clinicians^3^. However, these reports also contain carefully curated information from highly trained domain-expert human pathologists in the form of diagnoses and descriptions of morphology. Extracting relevant data from pathology reports can provide valuable insights, revealing disease patterns, treatment outcomes, and overall patient health^4^. Utilizing this information can lead to more accurate prognosis predictions and the creation of personalized treatment plans^5,6^. Beyond clinical use, this data is also essential for researchers constructing large-scale datasets and conducting medical studies.

Early work in this domain focused on developing ontology-driven applications for cancer type classification and staging. One study demonstrated the use of the National Cancer Institute (NCI) Thesaurus as a foundational ontological resource and developed a specialized ontology for the classification of glioma tumors^7^. Another notable effort employed organ-specific ontologies comprising axioms, logical rules, and structured classes to enable a formal representation of pathology reports, facilitating the prediction of TNM staging^8^.

Recent studies have demonstrated that large language models (LLMs) exhibit exceptional capabilities in leveraging their vast knowledge for tasks such as information extraction and certain forms of reasoning^9,10^. In the clinical domain, LLMs have shown remarkable potential, excelling at extracting data from medical texts without requiring extensive pretraining^11^. They have also proven highly effective in tasks like diagnosing complex medical cases^12–14^, mining radiology reports for key insights^15–18^, and extracting information from electronic health records^19–21^.

Despite these advances, the application of LLMs in natural language processing (NLP) tasks specific to pathology remains underexplored. A recent study employed a BERT-based model^22^ for named entity recognition, focusing on extracting diagnostic elements from breast pathology reports. However, this approach was limited to a single specimen class and lacked generalizability to other organ systems and disease processes^23^. More recently, large-scale LLMs like GPT have shown superior performance compared to supervised-trained models in various information extraction tasks^24^. Notably, GPT-4 was found to outperform supervised models in a zero-shot inference setting, without requiring additional training, although this study was also limited to breast cancer pathology reports.

In another study^25^, LLMs were used to estimate the TNM stage of cancers from pathology reports, a critical task for determining prognosis and guiding treatment decisions. However, cancer staging is not always systematically included in pathology reports, and previous methods for extracting TNM attributes relied on regular expressions or traditional NLP techniques. This study introduced a dedicated TNM staging LLM specifically designed for this task. Despite its utility, it does not provide comprehensive information about the anatomic extent of the cancer beyond tumor size, nodal involvement, and metastasis. As a result, relying solely on TNM staging may not fully capture the complexity of disease progression.

Moreover, these models are typically designed to handle a single task. Although they can be deployed locally, this setup may become expensive, as separate models are required to determine the tumor, nodes, and metastasis stages, respectively. An evaluation of the same task using open-source LLMs, combined with empirical tweaks to prompting techniques, assessed their performance in TNM staging^26^. However, a key limitation of these studies is that they tested their methodologies on extremely large models that require multiple GPUs with substantial VRAM, which is impractical for large-scale data mining, especially in resource-constrained settings. Furthermore, token generation with these large models is notably slow, further hindering their widespread application.

To address the issues mentioned above, we first evaluate the capabilities of both large-scale closed-source LLMs and open-source LLMs in performing information extraction tasks, such as identifying cancer types. Next, we assess their ability to holistically deduce the severity of cancer from pathology reports and predict the AJCC cancer stage. This staging system provides a more comprehensive assessment than the TNM stage, as it focuses beyond the anatomic extent of the cancer. Finally, we benchmark the performance of these LLMs in predicting patient prognosis from pathology reports alone, as a measure of performance on a complex reasoning task which requires both concrete information extraction and high-level reasoning.

After evaluating the performance of existing models, we developed a methodology to instruction-tune two new models that perform equally well, if not better, than both large-scale closed-source and open-source LLMs across all three tasks. The primary motivation for instruction tuning was to create a smaller, more efficient model capable of delivering high performance while being deployable in resource-constrained environments. We instruction-tuned GPT-4o-mini using a black-box fine-tuning approach provided by the OpenAI Platform, and Llama3.1-instruct-8B, which can be trained in an open-source environment. Notably, the Llama3.1-instruct-8B model, which we refer to as **Path-llama3.1-8B**, is a low-resource LLM capable of handling all three tasks efficiently and can be deployed in resource-constrained settings.

The key contributions of our study are as follows:

1. We conduct a comprehensive evaluation of both open-source (Llama3-Instruct 8B and Llama3-Instruct 70B)^27^ and closed-source LLMs (GPT-4o, GPT-4o-mini, Mistral-Large, Mistral-Medium)^28,29^ in zero-shot settings, focusing on information extraction tasks such as cancer type identification, as well as reasoning tasks like estimating the AJCC stage and predicting patient prognosis from pathology reports.
2. To enhance the reliability and accuracy of predictions, we developed our own instruction-tuned models that perform as well as, or better than, existing models. Notably, one of these is an open-source model that can deliver accurate predictions even in low-resource environments.
3. We open-source all code and trained models, making them easily deployable. Additionally, we provide the code required for further instruction tuning to adapt to hospital-specific scenarios, ensuring privacy and adaptability.

Our proposed methodology addresses the limitations of previous approaches, which primarily focus on developing cancer-specific TNM staging classifiers. In contrast, we aim to evaluate and benchmark the broader capabilities of large language models (LLMs) in clinical reasoning and the extraction of basic clinical information. A key distinction of our work lies in the use of a unified model for disease identification, AJCC cancer type classification, and prognosis assessment, whereas prior studies typically design separate models tailored to each task and cancer type, with an emphasis on low-level classifications that are later combined to produce high-level reasoning predictions. Additionally, to the best of our knowledge, we are the first to formulate a complete workflow for instruction tuning LLMs specifically for pathology-related information extraction. This framework provides a practical pathway for future researchers to instruction-tune models without the need to develop task-specific machine learning architectures. Instead, researchers can focus on curating high-quality question-and-answer pairs, significantly lowering the barrier to entry and reducing the complexity associated with model design and component-level optimization, which can otherwise have a substantial impact on performance.

## Results

To evaluate the performance of LLMs in cancer type identification, AJCC stage determination, and prognosis assessment, we begin by curating a custom dataset, the specifics of which are outlined in Section 4. The dataset is structured into three question-and-answer tasks, each addressing a specific aspect: cancer type identification, stage classification, and prognosis assessment. In each task, the question combines a query related to the respective topic cancer type, stage, or prognosis with the corresponding pathology report, and the answer represents the desired outcome. Given the verbosity of chat-based LLMs, we constrain their outputs to strictly adhere to a JSON object format^30^, structured as key-value pairs. This approach facilitates answer extraction using regular expressions for evaluation purposes while ensuring the outputs are well-suited for subsequent data mining and information extraction tasks.

For each task, we evaluate the performance of six models: two OpenAI models (GPT-4o and GPT-4o-mini)^28^, two Mistral models (Mistral-Medium and Mistral-Large)^29^, and two Llama 3 models (the 8B and 70B variants)^27^. Each experiment is conducted five times per model to ensure robust interpretations and to compute reliable confidence intervals. We perform instruction tuning by generating synthetic question-answer pairs, which, when combined with pathology reports, form a diverse and comprehensive training dataset. The** Path-GPT-4o-mini-FT** model is fine-tuned using OpenAI s proprietary platform, while **Path-llama3.1-8B** undergoes fine-tuning via Low-Rank Adaptation (LoRA)^31^, specifically optimizing key attention components. More details on data curation and model training are provided in Section 4.

### Cancer type identification

The six language models and two instruction-tuned models were evaluated on a test set comprising 952 pathology reports. These reports represent 32 distinct cancer types from various human body systems, providing a diverse and comprehensive dataset for evaluation. All eight models were assessed under identical system conditions and standardized user prompts, with outputs strictly formatted as JSON objects. The evaluation followed a zero-shot answering mechanism, as illustrated in Fig. 9A, with further details of the prompts provided in Appendix 1, Fig. 10.

**Fig. 1:**
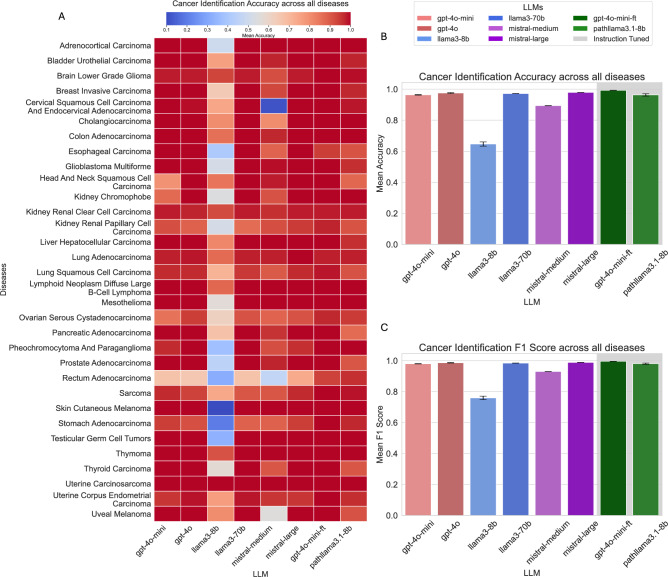
(**A**) Heatmap illustrating cancer identification accuracy across all diseases. (**B**) Cancer identification accuracy metrics. (**C**) Cancer identification F1-score metrics.

As depicted in Fig. 1, most language models demonstrated strong performance in cancer type identification, achieving a mean accuracy exceeding 96%, with the exceptions of Llama3-8B and Mistral-Medium. While Mistral-Medium achieved an accuracy of 90%, the Llama3-8B model performed significantly worse, with a mean accuracy of 64%. This underperformance is largely attributed to the model’s smaller parameter size and limited capability to follow instructions in tasks requiring information extraction from pathology text reports.

The fine-tuned GPT-4o-mini model, referred to as **Path-GPT-4o-mini-FT**, outperformed all other large language models, achieving a mean accuracy of 99%. This improvement is credited to its training on the instruction-tuning dataset, which not only enhanced its accuracy but also ensured consistent adherence to the JSON object output format. Similarly, the instruction-tuned Llama3.1 model, named **Path-llama3.1-8B**, exhibited a notable performance boost when fine-tuned on the same dataset, achieving a mean accuracy of 96%. This marks a substantial improvement for a model with only 8 billion parameters, highlighting the effectiveness of the fine-tuning process. The performance metrics by cancer type can be found in Appendix 2, Table 2.

Cancer type identification, being primarily an information extraction task, is the easiest of the three tasks. All LLMs exhibit high performance, with the exception of Llama3-8B, which struggles due to its smaller capacity and limited instruction-following capabilities. While Llama3-8B may successfully extract the correct cancer type, it fails to meet our strict evaluation criteria, designed with software deployment in mind, as it often produces outputs that deviate from the required format. However, with instruction tuning, the same-sized model, **Path-llama3.1-8B**, performs significantly better. This improvement is because the model learns to produce the exact output format required, making it more suitable for deployment in downstream tasks.

The other models perform well during inference, likely due to their robust pretraining on extensive and diverse datasets, which may include pathology-related text. Furthermore, these models have undergone instruction tuning through various chat-tuning frameworks that optimize their response generation using human feedback^32,33^. This fine-tuning process significantly enhances their ability to provide accurate, context-aware, and user-aligned responses, including outputs formatted as JSON objects, as required in this task.

**Fig. 2:**
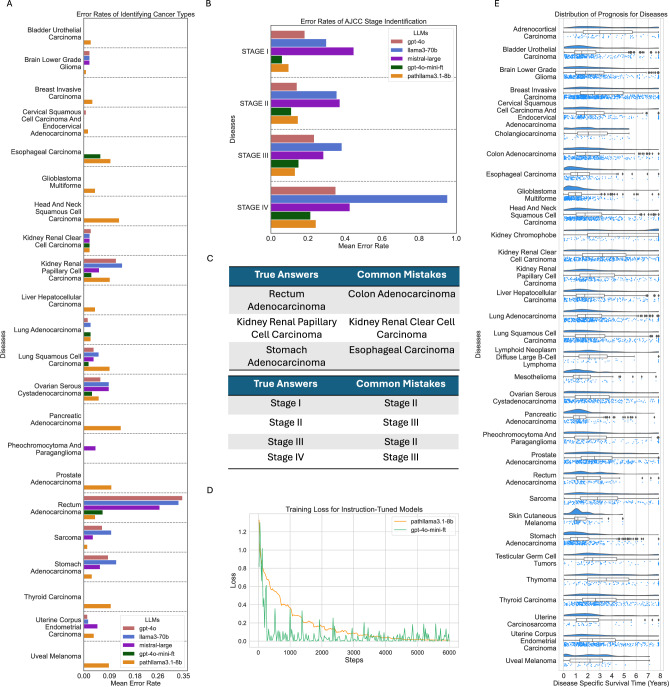
(**A**) Error rates of identifying cancer types. (**B**) Error rates of identifying AJCC Stage. (**C**) Common mistakes made by LLMs. (**D**) Training loss curves for the instruction-tuned models, plotted only for 6000 steps, although GPT-4o-mini was instruction-tuned for 17,000 steps. (**E**) Distribution of Disease-Specific Survival Time in years.

From Fig. 2, it is evident that the errors made by the LLMs are relatively straightforward and follow similar patterns across models. Figure 2A highlights the most common diseases and their corresponding error rates for both larger models and instruction-tuned models. A key observation is that all models tend to exhibit similar behavior when making mistakes, often confusing anatomically or functionally related organs, leading to incorrect predictions. For example, as shown in Fig. 2C, Rectum Adenocarcinoma is frequently misclassified as Colon Adenocarcinoma (The distinction between colon and rectal adenocarcinoma remains debated, as they are frequently classified together under the term **colorectal adenocarcinoma**). Similarly, confusion arises between Stomach Adenocarcinoma and Esophageal Carcinoma, reflecting the challenge of distinguishing closely related organ-specific cancers. Another notable misclassification occurs between Kidney Renal Papillary Cell Carcinoma and Kidney Renal Clear Cell Carcinoma. This further highlights how LLMs can struggle to distinguish between different cell types, leading to misclassifications. However, instruction tuning significantly reduces these error rates, demonstrating its effectiveness in improving the models’ ability to handle subtle distinctions and deliver more accurate predictions.

### AJCC stage identification

The same set of models was also evaluated on a dataset of 594 pathology reports to determine their corresponding stage (Stage I, II, III, or IV). These reports represent 21 distinct cancer types. The evaluation conditions were identical to those used for cancer type identification, with one key modification: a self-generated chain-of-thought query was included in the system prompt to guide the model through a step-by-step reasoning process to arrive at a specific answer, as shown in Fig. 9B. The full prompt is provided in Appendix 1, Fig. 11. While this approach increased both the input and output token counts, it significantly improved performance. However, the instruction-tuned models did not utilize the chain-of-thought methodology and instead adhered to a zero-shot answering mechanism.

**Fig. 3:**
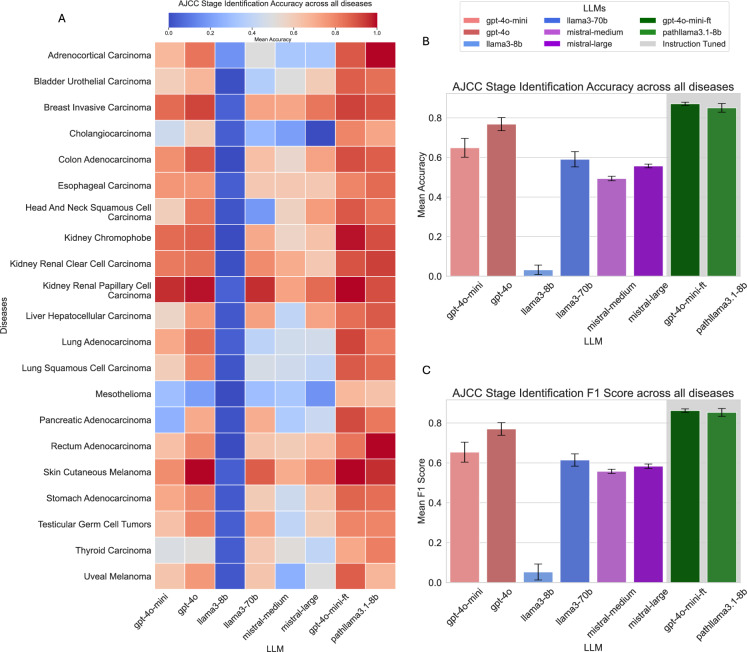
(**A**) Heatmap illustrating AJCC stage identification accuracy across all diseases. (**B**) AJCC stage identification accuracy metrics. (**C**) AJCC stage identification F1-score metrics.

As illustrated in Fig. 3, the performance of all LLMs is notably lower for AJCC stage identification compared to their results for cancer type identification. This discrepancy arises because stage estimation involves deductive reasoning, requiring not only insights from the provided text but also some level of world knowledge to make an informed prediction. A particularly notable outcome is the significant drop in performance of the Mistral models, which had performed well in the previous task. Similarly, the Llama3-8B model performs poorly, often deviating from the expected answer by producing irrelevant outputs, such as TNM staging information. Among the non-instruction-tuned models, OpenAI’s GPT-4o achieves the highest mean accuracy, reaching 76%.

The instruction-tuned models, however, demonstrate remarkable performance compared to running standard inference with LLMs. The **Path-GPT-4o-mini-FT** model achieves a mean accuracy of 87%, while the **Path-llama3.1-8B** model attains a mean accuracy of 85%. Notably, these results are achieved without employing a chain-of-thought approach. It is particularly significant that the **Path-llama3.1-8B**, despite being an 8B model, outperforms GPT-4o on this task. This underscores the potential of smaller, fine-tuned models to achieve high performance on specialized tasks, even surpassing larger, closed-source LLMs in certain scenarios. The performance metrics by cancer type can be found in Appendix 2, Table 3.

Determining the AJCC stage is inherently more challenging. In our initial experiments, we observed that when using a zero-shot approach, LLMs often default to predicting either Stage I or Stage IV. We speculate that this behavior stems from the training data these models are exposed to, which predominantly consists of internet text. Such data often lacks detailed clinical specifics and tends to portray cancer as a life-threatening condition, leading the models to overpredict Stage IV. Conversely, predicting Stage I may serve as a precautionary default response in the absence of sufficient context. In another experiment, we explored a two-step zero-shot prompting mechanism. In this approach, the LLMs were first asked to output the TNM stage, followed by determining the AJCC stage based on the provided TNM information. However, this method proved ineffective due to excessive hallucinations by the LLMs during the initial step, where the models often generated inaccurate or inconsistent TNM stage information, ultimately compromising the reliability of the subsequent AJCC stage prediction.

**Fig. 4:**
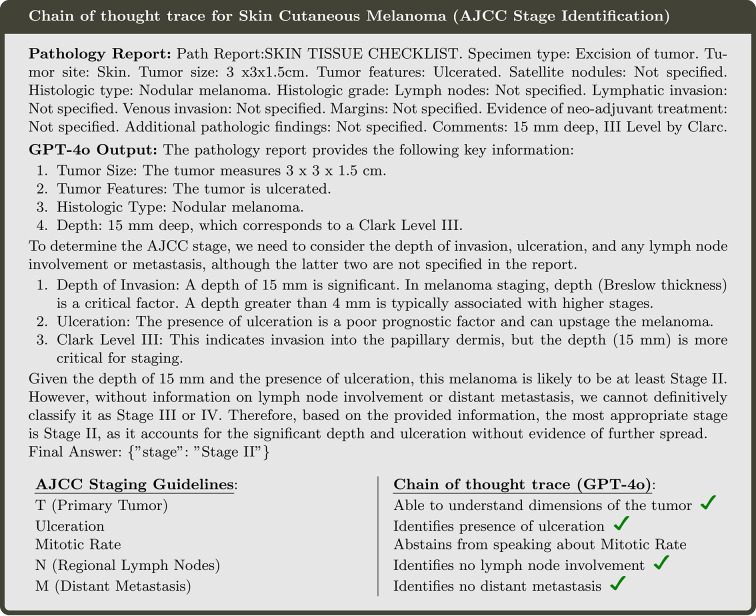
Example of how GPT-4o performs using Chain of thought in comparison to the standard AJCC Guidelines for Skin Cutaneous Melanoma.

To address the limitations observed in earlier experiments, we next explored an empirical approach that encouraged LLMs to implicitly reason and leverage their world knowledge. This was achieved by employing self-generated chain-of-thought (CoT) reasoning. As shown in Fig. 4, an LLM successfully creates a reasoning trace for Skin Cutaneous Melanoma that aligns closely with the AJCC staging guidelines. For instance, GPT-4o systematically extracts tumor size features, evaluates the spread of cancer to lymph nodes, and examines the presence of distant metastases. Moreover, it accurately identifies ulceration, a critical feature that can elevate the AJCC stage classification. In this particular example (Fig. 4), the model avoids hallucination, refraining from discussing features like the mitotic rate, which are absent from the report. This demonstrates the potential of CoT reasoning to improve the reliability and accuracy of complex staging tasks.

**Fig. 5:**
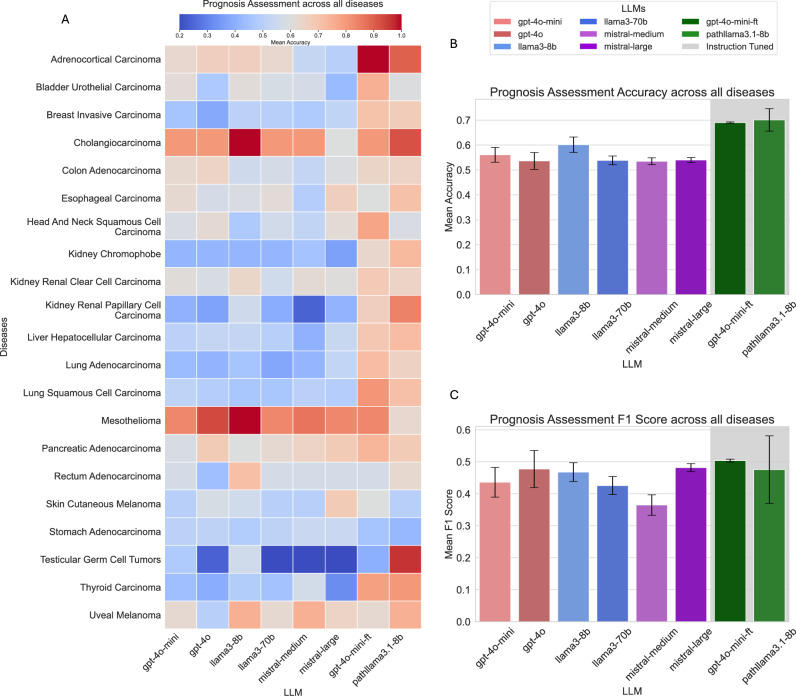
(**A**) Heatmap illustrating prognosis assessment accuracy across all diseases. (**B**) Prognosis assessment accuracy metrics. (**C**) Prognosis assessment F1-score metrics.

Upon analyzing certain outputs generated using the CoT methodology, we observed that some incorrect predictions were artifacts of misinterpreting specific terms within the context of staging. For example, the term “metastasis”, when mentioned in a context not directly referring to the patient’s current condition, was occasionally misinterpreted by the model as indicative of distant spread, leading to an erroneous classification of Stage IV (Fig. 5).

While chain-of-thought (CoT) reasoning certainly enhances performance and holds considerable potential for zero-shot inference, our instruction-tuned models demonstrate significantly better results. Instruction tuning aligns the model’s internal representations to interpret what a specific stage signifies in the context of a pathology report. This targeted alignment enables even an 8B parameter model, which requires minimal hardware (Can be trained on a NVIDIA 40x Series GPUs) for execution, to achieve high accuracy and F1 scores, highlighting the efficiency and practicality of instruction-tuned models for such tasks.

The mistakes observed in this task are particularly intriguing. Across all models, the average error rates are fairly similar, with the exception of Stage IV, where the error rate is slightly higher. A common pattern in the errors is that stages are often mispredicted as being one stage higher or lower than the correct stage. This suggests that while the models grasp the general progression of staging, they sometimes struggle with the finer distinctions required to accurately assign the correct AJCC stage.

**Fig. 6:**
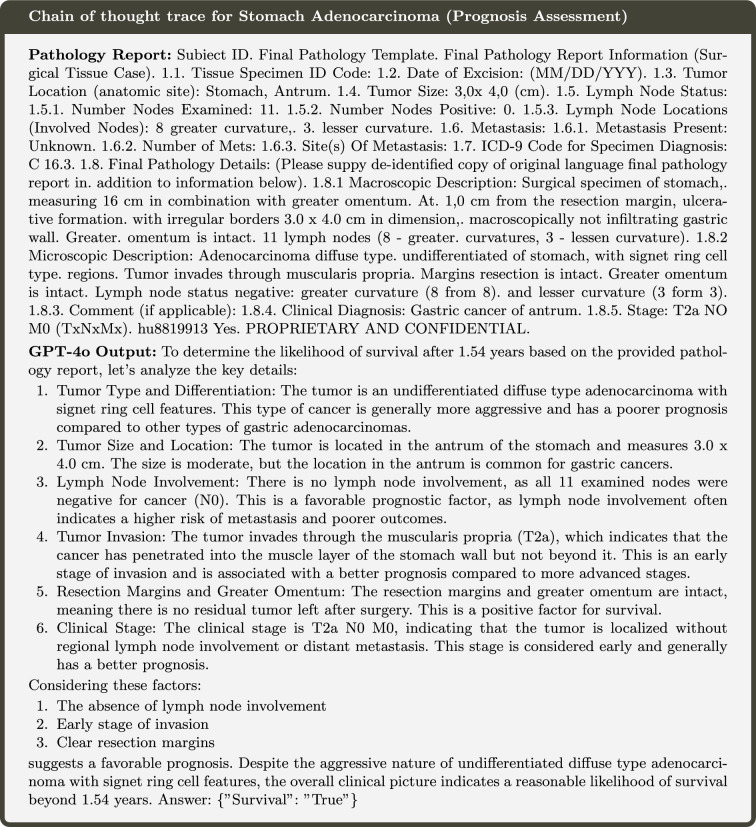
Example of how GPT-4o performs using Chain of thought for Prognosis Assessment.

From Fig. 2B,C, we observe that Stages II and III are often confused with one another, likely due to ambiguous interpretations of lymph node involvement in relation to the cancer. While Stage IV has strict guidelines requiring the presence of distant metastases for classification, it is notable that many instances are incorrectly classified as Stage III. This discrepancy warrants further investigation to better understand the reasoning behind such errors and to identify potential gaps in the models comprehension of AJCC staging criteria (Fig. 6).

### Prognosis assessment

For prognosis assessment, the task is formulated as a binary classification problem (as shown in Fig. 9C). The task is to predict whether a patient will survive beyond the disease-specific *mean survival time* based on the input pathology report. LLMs are provided with 8 examples (i.e., 8—consisting of 4 positive and 4 negative instances. Additionally, a self-generated chain-of-thought query is incorporated into the system prompt to guide the reasoning process. The full prompt is provided in Appendix 1, Fig. 12.

The models were evaluated on 593 pathology reports to assess prognosis, with the dataset encompassing 21 distinct cancer types. Among the three tasks, prognosis assessment proves to be the most challenging, as it involves a two-step process requiring both deductive reasoning and future outcome prediction. As shown in Fig. 5, notably, the Llama3-8B model achieves the highest accuracy; however, for this task, the F1 score provides a more comprehensive evaluation metric due to binary class imbalance. GPT-4o and Mistral-Large demonstrate higher F1 scores, primarily due to their better handling of the minority class in the prediction problem. Despite these observations, the task remains inherently difficult, as most models struggled to consistently produce correct answers.

Once again, the instruction-tuned models demonstrate superior performance, achieving both higher accuracy and higher F1 scores. Notably, the **Path-GPT-4o-mini-FT** model achieves the highest accuracy as well as the highest mean F1 score (The performance metrics by cancer type can be found in Appendix 2, Table 4.). It is particularly significant that this performance is attained without employing chain-of-thought reasoning or the 8-shot approach, highlighting the effectiveness of instruction tuning in enhancing model performance for this complex task.

Analyzing the performance of LLMs for prognosis is the most challenging of the three tasks. Even framing the problem required considerable effort, as estimating the number of years a patient is expected to survive based on a pathology report inherently resembles a regression problem. However, LLMs are notoriously poor at such tasks due to their lack of statistical understanding and inability to effectively interpret the complex interplay of features required for accurate prognosis estimation. This limitation highlights the need for alternative approaches to better leverage LLM capabilities for this problem domain.

To address the challenges, the problem was reframed by incorporating the disease-specific survival time into the dataset, providing the LLM with a virtual threshold to determine whether a patient would survive beyond that time. Additionally, we observed that relying solely on self-generated chain-of-thought reasoning was insufficient for achieving reliable results. To enhance performance, we provided a few example cases as references, enabling the model to better understand the task and produce more accurate answers. Despite these adjustments in methodology, most LLMs still fail to perform at a satisfactory level, indicating significant room for improvement. This underscores the inherent complexity of the prognosis task and highlights the limitations of current LLM capabilities in accurately addressing such nuanced problems.

**Fig. 7:**
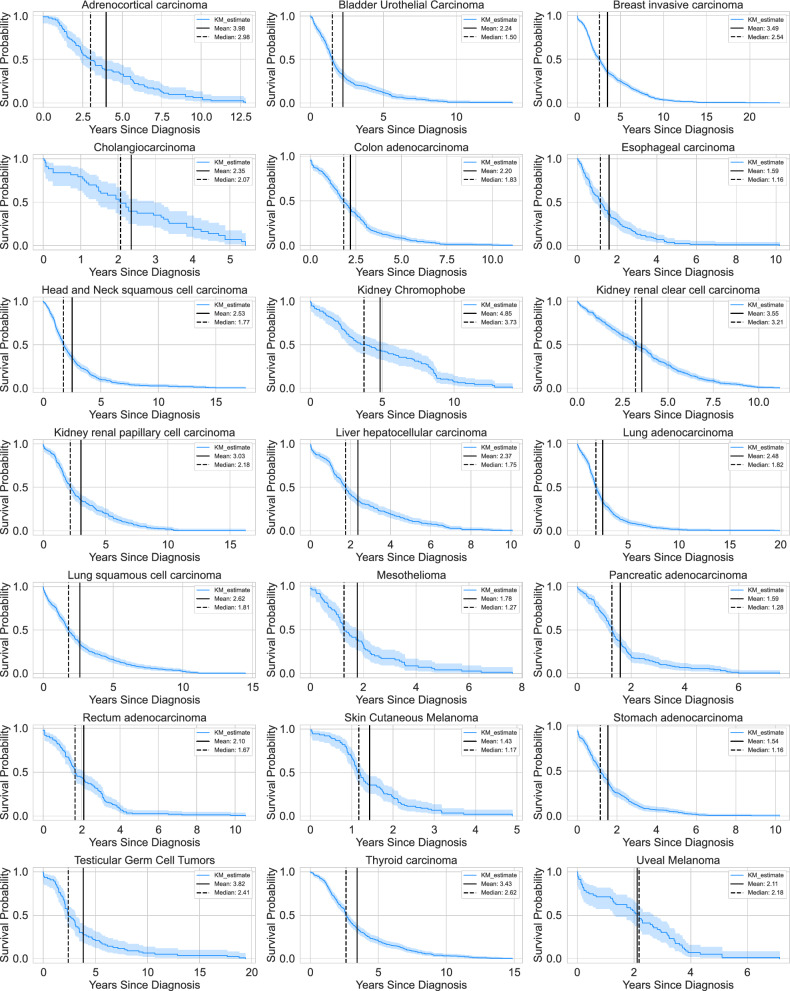
Kaplan-Meier curves for all cancer types for prognosis assessment.

From Fig. 2E and the Kaplan-Meier curves (Fig. 7), it is evident that the majority of cancer types included in our analysis have a mean survival time ranging between 1 and 4 years. Incorporating this information into the prompt enhanced the LLM’s understanding of disease severity, thereby improving its ability to analyze and predict prognosis more effectively. As illustrated in Fig. 6, GPT-4o systematically evaluates all relevant factors for Stomach Adenocarcinoma by constructing a rubric to identify positive and negative indicators for prognosis. The model effectively analyzes the pathology report, considering key elements such as lymph node involvement. For instance, it correctly concludes that the absence of lymph node involvement is a favorable indicator for the patient. Additionally, it examines resection margins and determines that since the margins are intact post-surgery, this also suggests a positive prognosis. This structured reasoning approach demonstrates LLMs’ capability to mimic clinical decision-making processes when provided with appropriate guidance.

### External validation

To assess the clinical applicability of our instruction-tuned model, we curated a set of 60 pathology reports from Weill Cornell Medicine, each annotated with the corresponding cancer type and AJCC stage, spanning six different cancer types (10 each). These reports were used as a fully out-of-distribution external validation set to simulate real-world hospital deployment. The model achieved 89% accuracy in cancer type identification and 70% accuracy in AJCC stage classification, demonstrating strong generalization to unseen clinical data (Table 1). The six cancer types were selected based on their relevance to gastrointestinal malignancies, a domain known for considerable ambiguity for cancer type identification in LLMs. The prognosis task was not evaluated on the external clinical dataset, as further research is required to advance its performance to a level suitable for clinical application. Achieving reliable and interpretable results in real-world settings remains an open challenge for future work.

**Table 1:** Performance metrics of our instruction-tuned model evaluated on pathology reports from Weill Cornell Medicine across six cancer types.

Cancer type	AJCC stage identification	Cancer type identification
Colon adenocarcinoma	0.7	0.8
Rectum adenocarcinoma	0.6	1.0
Pancreatic adenocarcinoma	0.6	1.0
Esophageal carcinoma	0.9	0.5
Cholangiocarcinoma	0.6	1.0
Liver hepatocellular carcinoma	0.8	1.0
Overall	0.7	0.89

The performance is accurately reflected when compared to the TCGA test set, with notable confusion observed between Esophageal Carcinoma and Stomach Adenocarcinoma, as well as between Colon Adenocarcinoma and Rectum Adenocarcinoma. However, for the remaining cancer types, our model achieves perfect performance. We observe a good out-of-distribution accuracy for AJCC stage identification as well, with the errors made by the instruction-tuned LLM aligning closely with those observed on the TCGA test set. The modest drop in performance on the external Weill Cornell dataset suggests that the model generalizes well, and importantly, this performance can be further improved through institution-specific instruction tuning. Our proposed methodology supports this form of adaptation, enabling seamless fine-tuning of the model to align with staging conventions and reporting styles unique to a particular hospital setting.

## Discussion

In this paper, we present a evaluation of the ability of recent large language models (LLMs) to assess cancer type, AJCC stage, and prognosis from pathology reports in a zero-shot setting. Our findings indicate that while LLMs excel in information extraction tasks, their performance declines in higher-level deduction-based reasoning tasks, such as determining cancer stage and prognosis. A potential enhancement to these tasks would be to reframe the first two problems cancer type identification and AJCC staging by incorporating multiple examples or shots within the prompt, similar to the approach used for the prognosis assessment task.

Additionally, we developed a robust evaluation framework that employs strict performance metrics and enforces a standardized output format, specifically JSON, facilitating eventual deployment in clinical settings. Furthermore, we present a methodology for instruction tuning LLMs, demonstrating that even smaller, open-source models can achieve performance on par with, or surpass, proprietary large LLMs across these three tasks.

Our analysis reveals that employing a self-generated chain-of-thought strategy significantly enhances model performance when used natively for inference. However, this approach can be computationally expensive during deployment due to the increased token usage in both inputs and outputs. Similarly, incorporating a few examples (or shots) in the system prompt improves predictions but suffers from the same limitation. Instruction tuning offers a practical solution by reducing inference time and computational costs, enabling hospitals to deploy specialized LLMs tailored for predefined tasks. These models are lightweight, require fewer resources, and are cost-effective to operate. Moreover, from a privacy standpoint, instruction-tuned models provide an additional advantage, as they can be deployed locally, ensuring that sensitive patient data is not shared with third-party services. To demonstrate the model’s effectiveness on real-world clinical pathology reports, we evaluated its performance on a set of 60 reports spanning six different cancer types. The instruction-tuned model exhibited good out-of-distribution performance, achieving high accuracy in both cancer type identification and AJCC stage classification, underscoring its potential for clinical deployment.

We also quantify the inference speed of our instruction-tuned model on an A100 GPU, achieving a throughput of approximately 267 tokens per second, which translates to processing a single pathology report in roughly 3 seconds. These performance metrics are based on real-world pathology reports extracted from clinical data, highlighting the model’s practical viability for deployment in time-sensitive healthcare environments.

While our research demonstrates the potential of LLMs in medical applications, there remain significant hurdles to overcome. One common issue is that LLMs often confuse textually similar diseases, highlighting the need for more robust system prompts to mitigate such errors. A more pressing challenge lies in estimating the AJCC stage, where the best observed performance is around 87%. Given the subjective nature of staging, establishing strict and standardized guidelines across diseases could facilitate large-scale data mining and improve model consistency in the future. In terms of prognosis assessment, while LLMs show promise, they are not yet ready for clinical deployment. Predicting a patient s prognosis requires a more holistic understanding of their clinical condition. Incorporating additional indicators and biomarkers, such as surgical history, molecular or genomic features (e.g., DNA sequencing results), and laboratory findings, would significantly enhance the model s predictive capabilities. Integrating these diverse data types into a multi-step data ingestion and reasoning process represents a promising direction for improving performance and achieving clinically actionable prognostic assessments. Integrating additional data sources, such as surgical records, laboratory results, and other electronic medical record (EMR) data, could significantly enhance their performance. This multimodal approach could provide a more comprehensive understanding of patient conditions, bringing LLMs closer to practical applications in clinical settings.

A next step in research, incorporating clinical data directly into the prompts could help LLMs make more informed and accurate decisions. An ambitious but impactful extension of our work would involve extracting chain-of-thought reasoning traces from pathologists and using this data for large-scale instruction tuning. Such an approach could significantly enhance the models’ ability to mimic expert reasoning, ultimately leading to higher performance metrics, particularly in challenging tasks like prognosis assessment.

To address erroneous AJCC stage classifications, future approaches could incorporate not only chain-of-thought reasoning traces handcrafted by pathologists but also domain-specific heuristics to guide model predictions more effectively. These additional heuristics may help the model better align clinical context with staging criteria, thereby improving overall performance. Furthermore, future iterations could integrate histopathology images alongside textual pathology reports. By combining image-based and text-based features, multimodal models may achieve more accurate and robust AJCC stage predictions.

Another important area for future improvement and evaluation is the performance of LLMs with respect to sensitive variables such as age, race, and sex. The dataset used in our analysis exhibits imbalances, particularly in age and racial representation, which may influence model behavior. Future research could focus on developing instruction-tuned models that are more robust and equitable across these demographic subgroups. Additionally, it would be valuable to investigate whether LLMs rely solely on token-level patterns to make predictions, or if they leverage deeper, internal representations of world knowledge when determining cancer type or stage. Understanding this distinction could offer insights into how these models reason about medical information and guide the development of more interpretable and trustworthy clinical AI systems.

As a future avenue of research, incorporating a retrieval-augmented generation (RAG) framework^34^ could significantly enhance the performance of open-source LLMs in these tasks. This approach would allow LLMs to access and reference hospital-specific guidelines, other pathology reports, and electronic medical record (EMR) texts, enabling them to make more informed decisions about staging and prognosis while maintaining privacy. Leveraging these diverse textual resources would provide the LLM with a broader and contextually richer knowledge base, improving its ability to generate accurate and reliable predictions. This strategy aligns with the goal of creating robust, specialized systems for clinical deployment.

In conclusion, LLMs demonstrate the potential to accurately identify cancer type, determine AJCC stage, and assess prognosis from pathology reports. In this study, we showcased various prompting methodologies and benchmarked both open-source and proprietary LLMs across a wide range of cancer types. Additionally, we developed two instruction-tuned models: one fine-tuned using OpenAI’s platform and another based on a 8B open-source LLM. The latter offers a resource-efficient and privacy-preserving solution, making it well-suited for deployment in clinical environments with minimal computational overhead.

## Methods

### Data source

We created the dataset used in this study by combining two publicly available datasets, both derived from The Cancer Genome Atlas (TCGA) initiative^35^. The TCGA-Reports dataset comprises the text of more than 9,000 pathology reports, extracted from the original PDF reports and cleaned into a machine-readable dataset^36^. The TCGA Pan-Cancer Clinical Data Resource (TCGA-CDR) contains clinical data including survival data for multiple outcomes measures^37^. These two datasets were merged using the unique sample-level TCGA barcodes. The final dataset comprised 9523 unique samples.

**Fig. 8:**
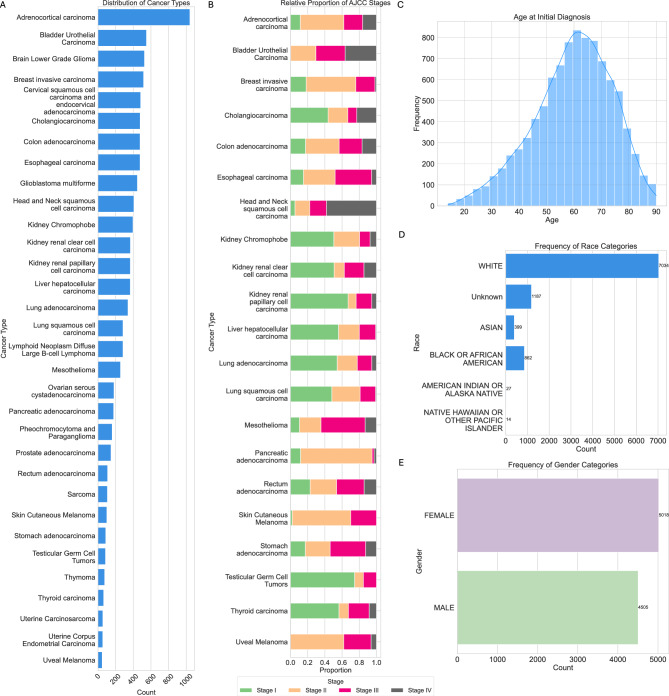
Summary overview of dataset. (**A**) Number of data points for each cancer type. (**B**) Distribution of AJCC stages for each cancer type where staging is present. (**C**) Distribution of age of patients at the time of diagnosis. (**D**) Distribution of race of patients. (**E**) Distribution of gender of patients.

From Fig. 8A, we observe the distribution of cancer types across the dataset, which includes 32 distinct types of cancers. For the cancer staging task, we considered only the high-level stage groups (e.g. Stage I, Stage II, etc.) and grouped sub-stages together. For example, Stage IIa and Stage IIb were both considered Stage II. Not all cancer types have stage data available, and relative frequencies of each stage vary between cancer types as shown in Fig. 8B.

For the prognosis task, disease-specific survival (DSS) was selected as the primary endpoint, with its distribution shown in Figure 2(E). DSS was chosen because pathology reports focus exclusively on the specimens examined, making DSS a more specific and relevant measure of prognosis for this disease process compared to broader metrics like overall survival. Prognostic categories were binarized based on the mean DSS within each cancer type to account for the significant variability in survival across different cancer types. This approach ensures that the classification aligns with the unique characteristics of each cancer type.

From Fig. 8C, we observe the distribution of age at initial diagnosis for all data points, with the mean age falling between 60 and 70 years. The dataset is skewed with respect to race but does include representation from minority groups, as shown in Fig. 8D. Additionally, the dataset is relatively balanced in terms of gender distribution, with a slightly higher number of female patients compared to male patients (Fig. 8E). The dataset was randomly split into training, validation, and testing sets according to an 80:10:10 ratio, stratified on cancer type to ensure similar class balance. We used 80% of the dataset to generate question answer pairs for instruction fine-tuning, while the remaining 20% was split equally, with 10% allocated for validation and 10% for testing. The same test set is used to evaluate both the direct inference capabilities of the LLMs and the performance of the instruction-tuned models, providing a consistent and fair benchmark for comparison across all methods. The complete data records are made public.

### Methodology for cancer type identification

All the LLMs, including the instruction-tuned models, were subjected to the same standardized prompt. Prompt engineering and selection, as noted in^38^, can be challenging and time-consuming, so we opted for a minimal and efficient approach. Each LLM was provided with both a system and a user prompt.

For the system prompt, we instructed the model to act as a highly knowledgeable pathology AI assistant, which helps align its internal representations and enhances its ability to generate accurate responses. Additionally, we constrained the model to select answers only from the cancer types present in the dataset. This restriction prevents the LLM from generating irrelevant cancer types and proves beneficial for consistent and effective data mining.

We also instructed the LLMs to avoid printing any extraneous text, addressing their inherent verbosity. Additionally, we explicitly directed the models not to fabricate information, thereby minimizing the likelihood of hallucination. Finally, we required the models to generate only the JSON object as the output (As of February 26, 2025, OpenAI provides structured output capabilities on its API platform. However, this feature is not yet available for open-source models), without providing any explanations or additional context, ensuring concise and standardized responses. An example of the prompt can be seen in Figure 10, in the Appendix.

### Methodology for AJCC stage identification

Identifying the AJCC stage is undoubtedly a more challenging task that requires a level of reasoning. Initially, attempts to have the LLMs predict the AJCC stage in a purely zero-shot setting were unsuccessful. Various prompting techniques were explored, including asking the models to first predict the TNM staging and then derive the AJCC stage, but these approaches also failed to yield accurate results. Ultimately, employing a self-generated chain-of-thought^39^ technique proved effective, enabling the LLMs to reason through the task and arrive at more accurate answers.

**Fig. 9:**
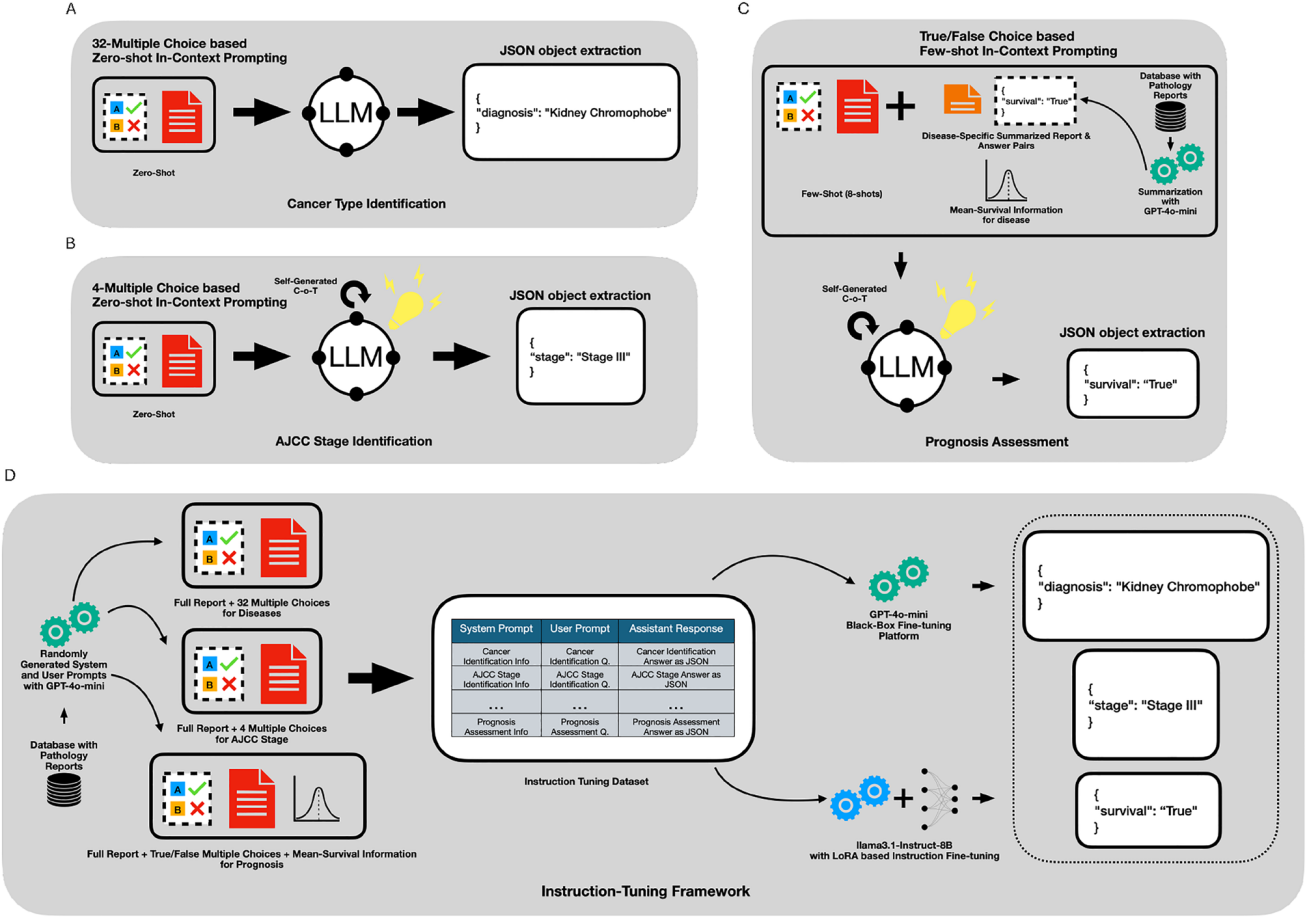
Summary overview of methodology. (**A**) Cancer Type Identification Methodology (**B**) AJJC Cancer Identification Methodology. (**C**) Prognosis Assessment Methodology (**D**) Instruction-tuning Methodology.

**Fig. 10:**
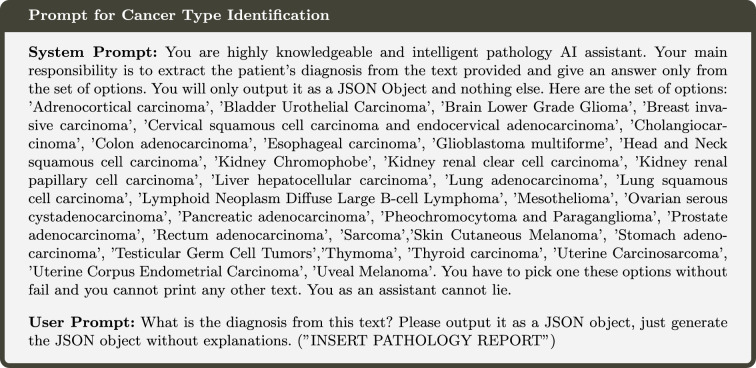
Prompt for cancer type identification.

**Fig. 11:**
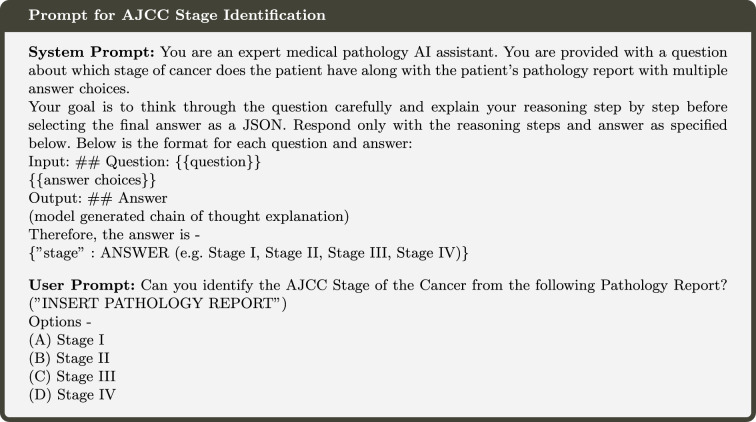
Prompt for AJCC stage identification.

From Fig. 9B, we observe that, similar to the cancer type identification task, a zero-shot prompting framework is employed, but with the addition of a self-generated chain-of-thought reasoning process that incorporates four AJCC stage options. As shown in Fig. 11 in the Appendix, we provide a well-structured template to guide the LLM in explaining its reasoning before presenting the final answer in JSON object format. Furthermore, the user prompt is restructured as a multiple-choice question, which has been shown to enhance performance by providing a clear decision framework for the model.

During inference, the system and user prompts for the instruction-tuned LLM do not include any chain-of-thought reasoning. Instead, the model is designed to operate in a purely zero-shot framework, directly generating outputs without any intermediate reasoning steps.

### Methodology for prognosis assessment

For the Prognosis Assessment task, similar to the stage identification task, a zero-shot prompting strategy alone proved ineffective, and even a zero-shot self-generated chain-of-thought approach failed to produce reliable results. To address this challenge, we restructured the problem by identifying a key limitation: the LLM lacked a reference point for distinguishing between good and poor prognoses for a given cancer type. While it may seem counterintuitive that large-scale LLMs lack this world knowledge, empirical observations suggest that they tend to predict extreme prognoses either overly optimistic or highly severe rather than producing a balanced assessment. To overcome this (as shown in Fig. 9C), we first extracted the mean disease-specific survival (DSS) time for each cancer type and included it in the prompt. Since DSS values provide a contextual anchor for prognosis estimation, similar summary statistics could also be sourced from the literature, ensuring that this approach remains applicable even when working with new datasets where the mean DSS is unknown. Additionally, we provided the LLM with four examples of good prognoses and four examples of poor prognoses, enabling it to better contextualize and evaluate prognosis predictions. In this context, a good prognosis is defined as a patient surviving beyond the mean disease-specific survival (DSS) time, while a bad prognosis is when survival falls below the mean DSS time. To further enhance the model’s performance, we also incorporated a self-generated chain-of-thought approach in the prompt. This strategy encourages the LLM to reason through the provided information systematically, improving its ability to make accurate prognosis predictions.

**Fig. 12:**
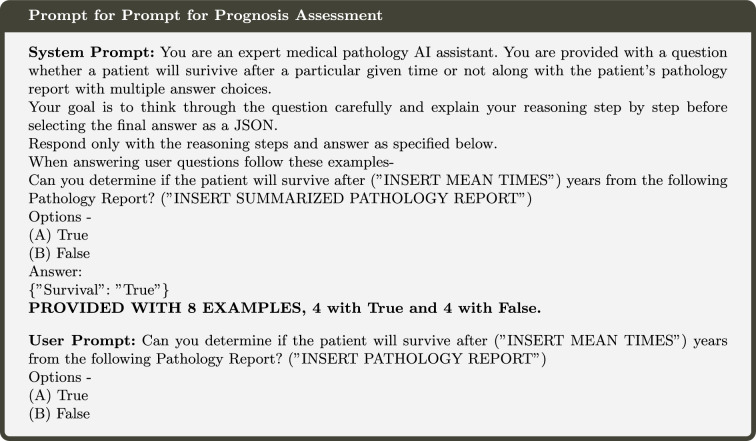
Prompt for prognosis assessment.

The few-shot framework presented challenges due to the length of the pathology reports, as including 8 full reports as examples often exceeded token limits and context length constraints. To address this issue, we summarized the reports using the GPT-4o-mini model, reducing their length while retaining essential information. These summaries were then included in the prompt, enabling the framework to remain within token limits while still providing relevant examples to guide the model’s predictions effectively. The example prompt can be seen in Fig. 12, in the Appendix.

During the evaluation or inference phase, the 8 examples (4 positive and 4 negative) are randomly selected from the training data and included in the prompt for guiding the model. However, when evaluating the instruction-tuned LLM, no chain-of-thought reasoning or few-shot examples are included. Instead, the instruction-tuned model operates in a pure zero-shot framework, with only the mean DSS time provided as context to support its predictions.

### Instruction-tuning framework

To build instruction-tuned models, we begin by synthetically generating question-and-answer pairs along with their corresponding system prompts. This is achieved by leveraging the training and validation datasets and using GPT-4o-mini to create textually diverse but semantically equivalent system and user prompts. These prompts are paired with the pathology reports and their respective answers. This approach produces a highly heterogeneous set of questions, allowing the LLM to be exposed to a broader range of variations during training. As a result, the model becomes more robust and better equipped to handle diverse queries in a clinical context.

To clarify, the content of the pathology reports remains unchanged; only the questions and system prompts are modified to avoid inducing incorrect hallucinations. These synthetically generated question-and-answer pairs are created for all three tasks using the training and validation datasets. This process results in a total of 17,344 question-and-answer pairs for training and 2,151 pairs for validation, providing a diverse and comprehensive dataset to effectively train the instruction-tuned models. The framework can be seen in Fig. 9D.

For the **Path-GPT-4o-mini-FT**, we directly utilize this dataset and fine-tune the model using the OpenAI Fine-Tuning platform. While the internal training strategy of the platform is not disclosed, we configured the training process with a learning rate multiplier of 0.3 and trained the model for approximately 17,000 steps. The number of steps was determined arbitrarily, as the internal mechanisms of OpenAI’s fine-tuning platform remain unknown. The convergence of the training loss curve is shown in Fig. 2D.

The training process for **Path-llama3.1-8B** involves specific considerations. We use the Llama3.1-8B-Instruct model as the base model for instruction tuning. Llama3.1 was chosen over Llama3 because, empirically, it demonstrates superior instruction-following capabilities due to extensive fine-tuning for instruction adherence, with a training emphasis on user-centric interactions. However, during direct inference and in-context prompting, Llama3 was observed to perform better on the tasks in this project. This is likely because Llama3 was originally trained as a general-purpose language model, offering more flexibility in specialized domains like pathology. In contrast, Llama3.1 incorporates more extensive instruction tuning aimed at general user-facing tasks, which may not align well with the nuanced and domain-specific nature of pathology-related information extraction.

To perform instruction tuning, we use Low-Rank Adaptation (LoRA)^31^ and fine-tune specific components of the model. Specifically, we adjust the Query Projection module to map the input embeddings into the query space and the Key Projection module to map the input embeddings into the key space. These adjustments are crucial for computing the attention weights within the LLM effectively. Additionally, we fine-tune the Value Projection module, which is responsible for extracting relevant information from the pathology reports.

Alongside these, we also fine-tune certain intermediate projectors to better align the model’s internal representations with the specific tasks and tune the Output Projection module to enhance task-specific outputs. This targeted approach allows us to adapt the model efficiently while preserving its overall capacity and ensuring it performs optimally for the given problem.

We use a LoRA rank of 16 and a LoRA alpha of 16 to implement rank-stabilized LoRA. These higher values were chosen because the task is particularly challenging, and this configuration was empirically determined to be optimal, balancing performance without leading to overfitting. A rank of 16 provides sufficient expressive capacity for instruction tuning to effectively adapt to pathology-related tasks. Lower ranks may fail to capture the nuanced complexity of pathology reports, while higher ranks could lead to increased computational overhead. An alpha value of 16 is appropriate, as it allows the model parameters to be adequately adjusted to the characteristics of this dataset, which differs substantially from the original training corpus used for Llama3.1-Instruct. To expedite training, we set the dropout rate to 0 and employed gradient checkpointing to reduce memory usage, enabling efficient fine-tuning on resource-constrained hardware.

The instruction tuning was conducted for 6000 steps, as shown in Fig. 2E, using a 4-bit quantized version of the Llama3.1-Instruct model. The maximum sequence length during training was set to 4,096 tokens to accommodate longer input contexts. The model was fine-tuned with a learning rate of $3e^{-4}$ using the *adamw_8bit* optimizer, which balances performance and memory efficiency, making it well-suited for resource-constrained training setups. The rest of training parameters are available in the code.

## Data Availability

The complete data set and code used to create it are available at - https://huggingface.co/datasets/rosenthal/tcga-path-notes.

## Appendix 1: Prompts

See Figs. 10, 11 and 12.

## Appendix 2: Performance metrics (extended)

See Tables 2, 3 and 4.

**Table 2:** Performance metrics by cancer type for GPT-4o and our instruction-tuned model, Pathllama3.1-8B, on the cancer type identification task.

Cancer types	GPT-4o		Pathllama3.1-8b	
Metrics	Accuracy	F1-score	Accuracy	F1-score
Adrenocortical carcinoma	1.00	1.00	1.00	1.00
Bladder urothelial carcinoma	1.00	1.00	0.97	0.99
Brain lower grade glioma	0.98	0.99	0.99	1.00
Breast invasive carcinoma	1.00	1.00	0.97	0.98
Cervical squamous cell carcinoma and endocervical adenocarcinoma	0.99	1.00	0.98	0.99
Cholangiocarcinoma	1.00	1.00	1.00	1.00
Colon adenocarcinoma	1.00	1.00	1.00	1.00
Esophageal carcinoma	1.00	1.00	0.91	0.95
Glioblastoma multiforme	1.00	1.00	0.96	0.98
Head and neck squamous cell carcinoma	1.00	1.00	0.88	0.93
Kidney chromophobe	1.00	1.00	1.00	1.00
Kidney renal clear cell carcinoma	0.97	0.99	0.98	0.99
Kidney renal papillary cell carcinoma	0.89	0.94	0.91	0.95
Liver hepatocellular carcinoma	1.00	1.00	0.96	0.98
Lung adenocarcinoma	0.99	0.99	0.98	0.99
Lung squamous cell carcinoma	0.96	0.98	0.91	0.95
Lymphoid neoplasm diffuse large B-cell lymphoma	1.00	1.00	1.00	1.00
Mesothelioma	1.00	1.00	1.00	1.00
Ovarian serous cystadenocarcinoma	0.94	0.97	0.95	0.97
Pancreatic adenocarcinoma	1.00	1.00	0.87	0.93
Pheochromocytoma and paraganglioma	1.00	1.00	1.00	1.00
Prostate adenocarcinoma	1.00	1.00	0.90	0.95
Rectum adenocarcinoma	0.65	0.79	0.96	0.98
Sarcoma	0.94	0.97	0.99	0.99
Skin cutaneous melanoma	1.00	1.00	1.00	1.00
Stomach adenocarcinoma	0.91	0.96	0.97	0.99
Testicular germ cell tumors	1.00	1.00	1.00	1.00
Thymoma	1.00	1.00	1.00	1.00
Thyroid carcinoma	1.00	1.00	0.90	0.95
Uterine carcinosarcoma	1.00	1.00	1.00	1.00
Uterine corpus endometrial carcinoma	0.99	0.99	0.96	0.98
Uveal melanoma	1.00	1.00	0.91	0.95
Overall	0.98	0.99	0.96	0.98

**Table 3:** Performance metrics by cancer type for GPT-4o and our instruction-tuned model, Pathllama3.1-8B, on the AJCC stage identification task.

Cancer types	GPT-4o		Pathllama3.1-8b	
Metrics	Accuracy	F1-score	Accuracy	F1-score
Adrenocortical carcinoma	0.83	0.83	1.00	1.00
Bladder urothelial carcinoma	0.68	0.66	0.84	0.84
Breast invasive carcinoma	0.93	0.93	0.90	0.90
Cholangiocarcinoma	0.60	0.48	0.72	0.68
Colon adenocarcinoma	0.89	0.88	0.88	0.88
Esophageal carcinoma	0.76	0.76	0.85	0.85
Head and neck squamous cell carcinoma	0.83	0.83	0.83	0.83
Kidney chromophobe	0.87	0.89	0.91	0.91
Kidney renal clear cell carcinoma	0.84	0.84	0.93	0.93
Kidney renal papillary cell carcinoma	0.99	0.99	0.91	0.95
Liver hepatocellular carcinoma	0.75	0.81	0.88	0.91
Lung adenocarcinoma	0.85	0.86	0.81	0.82
Lung squamous cell carcinoma	0.79	0.80	0.85	0.88
Mesothelioma	0.20	0.18	0.63	0.58
Pancreatic adenocarcinoma	0.70	0.79	0.83	0.87
Rectum adenocarcinoma	0.79	0.83	1.00	1.00
Skin cutaneous melanoma	1.00	1.00	0.96	0.96
Stomach adenocarcinoma	0.79	0.79	0.85	0.84
Testicular germ cell tumors	0.80	0.71	0.80	0.71
Thyroid carcinoma	0.50	0.56	0.81	0.82
Uveal melanoma	0.75	0.74	0.68	0.74
Overall	0.77	0.77	0.85	0.85

**Table 4:** Performance metrics by cancer type for GPT-4o and our instruction-tuned model, Pathllama3.1-8B, on the Prognosis Assessment task.

Cancer types	GPT-4o		Pathllama3.1-8b	
Metrics	Accuracy	F1-score	Accuracy	F1-score
Adrenocortical carcinoma	0.67	0.50	0.90	0.53
Bladder urothelial carcinoma	0.47	0.47	0.59	0.35
Breast invasive carcinoma	0.39	0.41	0.68	0.43
Cholangiocarcinoma	0.80	0.00	0.92	0.60
Colon adenocarcinoma	0.65	0.68	0.65	0.52
Esophageal carcinoma	0.57	0.56	0.71	0.60
Head and neck squamous cell carcinoma	0.62	0.62	0.59	0.50
Kidney chromophobe	0.42	0.48	0.73	0.59
Kidney renal clear cell carcinoma	0.58	0.67	0.65	0.63
Kidney renal papillary cell carcinoma	0.37	0.45	0.84	0.75
Liver hepatocellular carcinoma	0.53	0.56	0.72	0.51
Lung adenocarcinoma	0.41	0.42	0.66	0.53
Lung squamous cell carcinoma	0.49	0.53	0.71	0.62
Mesothelioma	0.93	0.60	0.63	0.00
Pancreatic adenocarcinoma	0.68	0.48	0.68	0.48
Rectum adenocarcinoma	0.44	0.56	0.63	0.61
Skin cutaneous melanoma	0.58	0.27	0.50	0.00
Stomach adenocarcinoma	0.51	0.59	0.42	0.30
Testicular germ cell tumors	0.24	0.35	0.96	0.93
Thyroid carcinoma	0.40	0.35	0.80	0.50
Uveal melanoma	0.50	0.50	0.75	0.00
Overall	0.54	0.48	0.70	0.48

## Code availability

The complete code and the models are available at - https://github.com/rachitsaluja/PathRep-Bench.

## References

[1] Siegel, R. L., Miller, K. D., Wagle, N. S. & Jemal, A. Cancer statistics, 2023. *CA Cancer J. Clin.* **73**(1), 17–48 (2023). 36633525 10.3322/caac.21763

[2] Swillens, J., Sluijter, C., Overbeek, L., Nagtegaal, I. & Hermens, R. Identification of barriers and facilitators in nationwide implementation of standardized structured reporting in pathology: a mixed method study. *Virchows Archiv.* **475**, 551–561 (2019). 31270615 10.1007/s00428-019-02609-6 PMC6861434

[3] Sluijter, C. E., van Lonkhuijzen, L. R., van Slooten, H.-J., Nagtegaal, I. D. & Overbeek, L. I. The effects of implementing synoptic pathology reporting in cancer diagnosis: A systematic review. *Virchows Archiv.* **468**(6), 639–649 (2016). 27097810 10.1007/s00428-016-1935-8 PMC4887530

[4] Truhn, D. et al. Extracting structured information from unstructured histopathology reports using generative pre-trained transformer 4 (gpt-4). *J. Pathol.* **262**(3), 310–319 (2024). 38098169 10.1002/path.6232

[5] Dumbrava, E. I. & Meric-Bernstam, F. Personalized cancer therapy-leveraging a knowledge base for clinical decision-making. *Mol. Case Stud.* **4**(2), a001578 (2018). 10.1101/mcs.a001578 PMC5880252 29212833

[6] Benary, M. et al. Leveraging large language models for decision support in personalized oncology. *JAMA Netw. Open* **6**(11), e2343689–e2343689 (2023). 37976064 10.1001/jamanetworkopen.2023.43689 PMC10656647

[7] Marquet, G., Dameron, O., Saikali, S., Mosser, J. & Burgun, A. Grading glioma tumors using owl-dl and nci thesaurus. in *AMIA Annual Symposium Proceedings*, vol. 2007, 508, (2007). PMC2655830 18693888

[8] Franca, F., Schulz, S., Bronsert, P., Novais, P. & Boeker, M. Feasibility of an ontology driven tumor-node-metastasis classifier application: A study on colorectal cancer. in *2015 International Symposium on Innovations in Intelligent SysTems and Applications (INISTA)*, pp. 1–7, IEEE, (2015).

[9] Brown, T., Mann, B., Ryder, N., Subbiah, M., Kaplan, J. D., Dhariwal, P., Neelakantan, A., Shyam, P., Sastry, G., Askell, A., Agarwal, S., Herbert-Voss, A., Krueger, G., Henighan, T., Child, R., Ramesh, A., Ziegler, D., Wu, J., Winter, C., Hesse, C., Chen, M., Sigler, E., Litwin, M., Gray, S., Chess, B., Clark, J., Berner, C., McCandlish, S., Radford, A., Sutskever, I. & Amodei, D. Language models are few-shot learners. in *Advances in Neural Information Processing Systems* (H. Larochelle, M. Ranzato, R. Hadsell, M. Balcan, and H. Lin, eds.), vol. 33, pp. 1877–1901, Curran Associates, Inc., (2020).

[10] Kojima, T., Gu, S. S., Reid, M., Matsuo, Y. & Iwasawa, Y. Large language models are zero-shot reasoners. *Adv. Neural Inform. Process. Syst.* **35**, 22199–22213 (2022).

[11] Agrawal, M., Hegselmann, S., Lang, H., Kim, Y. & Sontag, D. Large language models are few-shot clinical information extractors. in *Proceedings of the 2022 Conference on Empirical Methods in Natural Language Processing* (Y. Goldberg, Z. Kozareva, and Y. Zhang, eds.), (Abu Dhabi, United Arab Emirates), pp. 1998–2022, Association for Computational Linguistics, Dec. (2022).

[12] Eriksen, A. V., Möller, S. & Ryg, J. Use of gpt-4 to diagnose complex clinical cases. *NEJM AI*, vol. 1, no. 1, p. AIp2300031, (2024).

[13] Barile, J. et al. Diagnostic accuracy of a large language model in pediatric case studies. *JAMA Pediatr.* **178**(3), 313–315 (2024). 38165685 10.1001/jamapediatrics.2023.5750 PMC10762631

[14] Goh, E., Gallo, R., Hom, J., Strong, E., Weng, Y., Kerman, H., Cool, J., Kanjee, Z., Parsons, A. S., Ahuja, N. *et al.* Influence of a large language model on diagnostic reasoning: A randomized clinical vignette study. *medRxiv*, (2024). 10.1001/jamanetworkopen.2024.40969 PMC11519755 39466245

[15] Fink, M. A. et al. Potential of chatgpt and gpt-4 for data mining of free-text ct reports on lung cancer. *Radiology* **308**(3), e231362 (2023). 37724963 10.1148/radiol.231362

[16] Liu, Q., Hyland, S., Bannur, S., Bouzid, K., Castro, D., Wetscherek, M., Tinn, R., Sharma, H., Pérez-García, F., Schwaighofer, A., Rajpurkar, P., Khanna, S., Poon, H., Usuyama, N., Thieme, A., Nori, A., Lungren, M., Oktay, O., Alvarez-Valle, J. Exploring the boundaries of GPT-4 in radiology. in *Proceedings of the 2023 Conference on Empirical Methods in Natural Language Processing* (H. Bouamor, J. Pino, and K. Bali, eds.), (Singapore), pp. 14414–14445, Association for Computational Linguistics, Dec. (2023).

[17] Mukherjee, P., Hou, B., Lanfredi, R. B. & Summers, R. M. Feasibility of using the privacy-preserving large language model vicuna for labeling radiology reports. *Radiology* **309**(1), e231147 (2023). 37815442 10.1148/radiol.231147 PMC10623189

[18] Horiuchi, D. et al. Accuracy of chatgpt generated diagnosis from patient’s medical history and imaging findings in neuroradiology cases. *Neuroradiology* **66**(1), 73–79 (2024). 37994939 10.1007/s00234-023-03252-4

[19] Yang, X. et al. A large language model for electronic health records. *NPJ Digital Med.* **5**(1), 194 (2022). 10.1038/s41746-022-00742-2 PMC9792464 36572766

[20] Luo, R. et al. Biogpt: Generative pre-trained transformer for biomedical text generation and mining. *Briefings Bioinform.* **23**(6), bbac409 (2022). 10.1093/bib/bbac409 36156661

[21] Guevara, M. et al. Large language models to identify social determinants of health in electronic health records. *NPJ Digital Med.* **7**(1), 6 (2024). 10.1038/s41746-023-00970-0 PMC10781957 38200151

[22] Devlin, J., Chang, M.-W., Lee, K. & Toutanova, K. BERT: Pre-training of deep bidirectional transformers for language understanding. in *Proceedings of the 2019 Conference of the North American Chapter of the Association for Computational Linguistics: Human Language Technologies, Volume 1 (Long and Short Papers)* (J. Burstein, C. Doran, and T. Solorio, eds.), (Minneapolis, Minnesota), pp. 4171–4186, Association for Computational Linguistics, June (2019).

[23] Zeng, K. G., Dutt, T., Witowski, J., Kiran, G. K., Yeung, F., Kim, M., Kim, J., Pleasure, M., Moczulski, C., Lopez, L. J. L. *et al.* Improving information extraction from pathology reports using named entity recognition. *Research Square*, (2023).

[24] Sushil, M., Zack, T., Mandair, D., Zheng, Z., Wali, A., Yu, Y.-N., Quan, Y., Lituiev, D. & Butte, A. J. A comparative study of large language model-based zero-shot inference and task-specific supervised classification of breast cancer pathology reports. *J. Am. Med. Inform. Assoc*. p. ocae146, (2024). 10.1093/jamia/ocae146 PMC11413420 38900207

[25] Kefeli, J., Berkowitz, J., Acitores Cortina, J. M., Tsang, K. K. & Tatonetti, N. P. Generalizable and automated classification of tnm stage from pathology reports with external validation. *Nat. Commun.* **15**(1), 8916 (2024). 39414770 10.1038/s41467-024-53190-9 PMC11484761

[26] Change, C.-H., Lucas, M. M., Lu-Yao, G. & Yang, C. C. Classifying cancer stage with open-source clinical large language models. in *2024 IEEE 12th International Conference on Healthcare Informatics (ICHI)*, pp. 76–82, IEEE, (2024).

[27] Dubey, A., Jauhri, A., Pandey, A., Kadian, A., Al-Dahle, A., Letman, A., Mathur, A., Schelten, A., Yang, A., Fan, A. *et al.* The llama 3 herd of models. *arXiv preprint* arXiv:2407.21783, (2024).

[28] Hurst, A., Lerer, A., Goucher, A. P., Perelman, A., Ramesh, A., Clark, A., Ostrow, A., Welihinda, A., Hayes, A., Radford, A., *et al.* Gpt-4o system card. *arXiv preprint* arXiv:2410.21276, (2024).

[29] Jiang, A. Q., Sablayrolles, A., Roux, A., Mensch, A., Savary, B., Bamford, C., Chaplot, D. S., Casas, D. d. l., Hanna, E. B., Bressand, F., *et al.* Mixtral of experts. *arXiv preprint* arXiv:2401.04088, (2024).

[30] Liu, M. X., Liu, F., Fiannaca, A. J., Koo, T., Dixon, L., Terry, M. & Cai, C. J. ” we need structured output”: Towards user-centered constraints on large language model output. in *Extended Abstracts of the CHI Conference on Human Factors in Computing Systems*, pp. 1–9, (2024).

[31] Hu, E. J. et al. Lora: Low-rank adaptation of large language models. *ICLR* **1**(2), 3 (2022).

[32] Rafailov, R., Sharma, A., Mitchell, E., Manning, C. D., Ermon, S. & Finn, C. Direct preference optimization: Your language model is secretly a reward model. *Adv. Neural Inform. Process. Syst*. **36** (2024).

[33] Ouyang, L. et al. Training language models to follow instructions with human feedback. *Adv. Neural Inform. Process. Syst.* **35**, 27730–27744 (2022).

[34] Lewis, P. et al. Retrieval-augmented generation for knowledge-intensive nlp tasks. *Adv. Neural Inform. Process. Syst.* **33**, 9459–9474 (2020).

[35] Weinstein, J. N. et al. The Cancer Genome Atlas Pan-Cancer analysis project. *Nat. Genet.* **45**, 1113–1120 (2013). 24071849 10.1038/ng.2764 PMC3919969

[36] Kefeli, J. & Tatonetti, N. TCGA-Reports: A machine-readable pathology report resource for benchmarking text-based AI models. *Patterns*, vol. 5, Mar. (2024). 10.1016/j.patter.2024.100933 PMC10935496 38487800

[37] Liu, J. et al. An integrated tcga pan-cancer clinical data resource to drive high-quality survival outcome analytics. *Cell* **173**(2), 400–416 (2018). 29625055 10.1016/j.cell.2018.02.052 PMC6066282

[38] Sahoo, P., Singh, A. K., Saha, S., Jain, V., Mondal, S. & Chadha, A. A systematic survey of prompt engineering in large language models: Techniques and applications. *arXiv preprint* arXiv:2402.07927, (2024).

[39] Wei, J. et al. Chain-of-thought prompting elicits reasoning in large language models. *Adv. Neural Inform. Process. Syst.* **35**, 24824–24837 (2022).

